# Redesign of the monomer–monomer interface of Cre recombinase yields an obligate heterotetrameric complex

**DOI:** 10.1093/nar/gkv901

**Published:** 2015-10-10

**Authors:** Chi Zhang, Connie A. Myers, Zongtai Qi, Robi D. Mitra, Joseph C. Corbo, James J. Havranek

**Affiliations:** 1 Program in Computational and Systems Biology, Washington University in St Louis, St Louis, MO 63110, USA; 2 Department of Pathology and Immunology, Washington University in St Louis, St Louis, MO 63110, USA; 3 Program in Molecular Genetics and Genomics, Washington University in St Louis, St Louis, MO 63110, USA; 4 Department of Genetics, Washington University in St Louis, St Louis, MO 63110, USA; 5 Department of Biochemistry and Molecular Biophysics, Washington University in St Louis, St Louis, MO 63110, USA

## Abstract

Cre recombinase catalyzes the cleavage and religation of DNA at loxP sites. The enzyme is a homotetramer in its functional state, and the symmetry of the protein complex enforces a pseudo-palindromic symmetry upon the loxP sequence. The Cre-lox system is a powerful tool for many researchers. However, broader application of the system is limited by the fixed sequence preferences of Cre, which are determined by both the direct DNA contacts and the homotetrameric arrangement of the Cre monomers. As a first step toward achieving recombination at arbitrary asymmetric target sites, we have broken the symmetry of the Cre tetramer assembly. Using a combination of computational and rational protein design, we have engineered an alternative interface between Cre monomers that is functional yet incompatible with the wild-type interface. Wild-type and engineered interface halves can be mixed to create two distinct Cre mutants, neither of which are functional in isolation, but which can form an active heterotetramer when combined. When these distinct mutants possess different DNA specificities, control over complex assembly directly discourages recombination at unwanted half-site combinations, enhancing the specificity of asymmetric site recombination. The engineered Cre mutants exhibit this assembly pattern in a variety of contexts, including mammalian cells.

## INTRODUCTION

**Figure 1.**
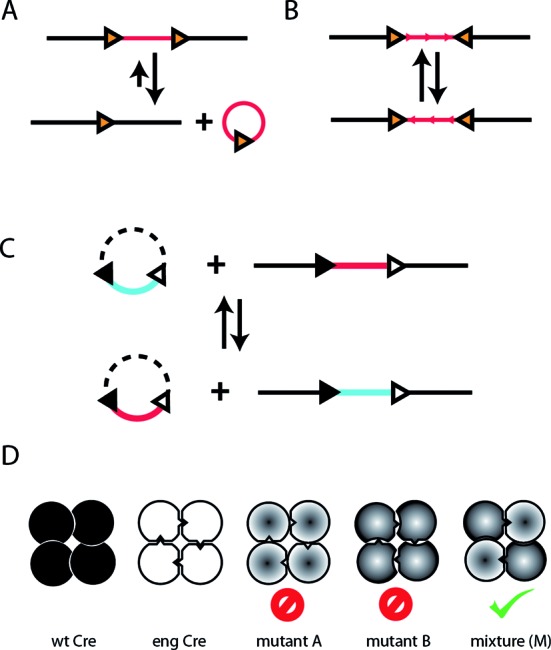
Genomic applications of Cre recombinase. Depending on the number and relative orientation of the loxP sites, Cre recombinase can perform deletion, inversion, insertion or exchange of genetic content. (**A**) Direct repeats of the loxP site can be recombined to excise the intervening genetic interval (downward arrow). This reaction is also catalyzed in the reverse direction, yielding a genetic insertion (upward arrow). For thermodynamic reasons, the excision reaction is favored and insertion events occur with low frequency. (**B**) Inverted loxP repeats can be recombined to yield an inversion of the bracketed DNA. (**C**) Recombination at pairs of distinct RT sites gives rise to exchange of the intervening genetic ‘cassette’. (**D**) Cre recombinase is a homotetramer in its functional complex (wt Cre), imparting a preference for a symmetric RT as a consequence. As a first step to achieving recombination at asymmetric sites, we desire an orthogonal engineered interface between Cre monomers (eng Cre). We seek to construct a novel homotetramer Cre mutant with monomer–monomer interfaces that, while functional, are incompatible with the wild-type protein. Combining wild-type and engineered half-interfaces gives rise to two distinct mutants that cannot form functional complexes (mutants A and B). Combining the two mutants (denoted by ‘M’) can reconstitute a functional heterotetrameric complex, which contains two wild-type and two engineered interfaces.

Cre recombinase forms a tetrameric complex that splices DNA molecules containing the 34-bp recombination target (RT) site loxP (1), recombining two DNA molecules in *trans* to accomplish an insertion or translocation event, or in *cis* to achieve either gene excision or inversion, depending on the relative orientation of the loxP sites (Figure 1). Cre recombinase has been used to generate conditional gene knockouts, where a gene of interest is flanked by loxP sites (‘floxed’) (2). Expression of Cre recombinase under the control of promoters that are specific for particular tissues or developmental stages abrogates gene function by physical excision from the genome. The utility of this system depends on the functional autonomy of Cre recombinase: the enzyme requires no other factors to splice DNA and is capable of modifying genomes in non-replicating cells, where the efficacy of gene conversion via double-strand break (DSB) induced homologous recombination is expected to be low (3,4).

Another application for Cre recombinase is recombination-mediated cassette exchange (RMCE) (5), also known as double-reciprocal crossover (6,7) or double-lox replacement (8,9). In this approach, (reviewed in ref. (10)) recombination between DNA molecules that share two neighboring heterologous RT sites accomplishes the exchange of the bounded genetic interval (the cassette) between the sites (Figure 1C). This has been demonstrated using both Flp and Cre recombinase with heterologous RT variants (5,8), as well as simultaneously with Cre and the Flp recombinases (11). Although RMCE has so far only been demonstrated with wild-type recombinase proteins and RT sites, the approach has many attractive features as a tool for genome engineering. First, it has a higher efficiency for gene conversion than does Cre-mediated insertion, as it does not require survival of insertional events that are susceptible to reversal by excision (8). Second, the cassettes that are exchanged are precisely demarcated, yielding truly ‘scarless’ genomic surgery. Third, the process requires less Cre protein than recombinational insertion, resulting in less cytotoxicity(8). Finally, the autonomy of Cre as a recombinase suggests that RMCE could prove to be effective in terminally differentiated cells, in contrast to strategies for gene conversion that rely upon homology directed repair.

One impediment to broader use of Cre recombinase is the inflexibility of the binding site specificity. In contrast to DNA binding proteins whose specificity derives from the assembly of small recognition modules such as zinc finger or TAL effector domains, Cre recombinase interacts with DNA through large interfaces that defy a modular decomposition. Nevertheless, altered RT specificities have been elicited in mutant Cre recombinases using directed evolution (12–14).

The quaternary structure of the Cre complex creates a second challenge for engineering novel RT specificities. The four-fold symmetry in the functional protein complex imposes a pseudo-palindromic symmetry upon the RT site. The loxP site consists of two 13 bp palindromic half-sites separated by an asymmetric 8 bp spacer that gives loxP its direction. The utility of targeting Cre mutants to altered RT sites is severely compromised if only pseudo-palindromic sites may be considered. This limitation has been addressed by using directed evolution to generate mutant homotetrameric complexes that can operate on asymmetric sites (14,15). However, requiring a single Cre mutant to operate on two different half-sites is likely to result in promiscuous enzymes. Separate Cre mutants with specificities toward the two half-sites of an asymmetric RT site may be able to recombine these sites, but the lack of control over assembly of the complex allows for any combination of these half-sites as potential sites for recombination (16). Some of these combinations will be undesired, generating off-target recombination events and exacerbating the cytotoxicity of Cre recombinase (17).

A similar technical challenge has been overcome in the design of zinc finger nucleases (ZFNs). ZFNs are DSB agents that achieve their sequence specificity by concatenating multiple zinc finger modules, each of which recognizes 3–4 bp. The cleavage activity is provided by the dimeric FokI nuclease. FokI monomers are genetically fused to zinc finger arrays, and two such constructs that converge upon a DNA site reconstitute a functional nuclease dimer, inducing a DSB. The development of obligate heterodimer FokI mutants has increased target specificity and reduced cytotoxicity in this system (18). Under this approach, the ZFNs that co-locate on desired cleavage sites must contribute two distinct FokI monomers; misassembly of two copies of the same ZFN at an off-target site cannot reconstitute a functional nuclease. Constructing a functional Cre complex from distinguishable and separately mutatable monomers is an attractive strategy for enhancing the specificity of RT site recognition. An earlier effort to generate heterotetramer Cre mutants succeeded in forming a novel functional interface, but one of the two mutants retained significant activity as a homotetramer (19).

In this manuscript we describe the engineering of Cre mutants that are inactive in isolation, but are functional as a (ABAB) heterotetramer when both mutants are present. We use a combination of computational and rational design to select mutations that are predicted to form a novel interface between Cre monomers that is functional, but whose halves are incompatible with their wild-type counterparts. We show that the negative engineering goal (incompatibility with wild-type) is more difficult to achieve than the positive goal (full functionality), requiring three iterations of mutation. The obligate heterotetrameric assembly of the pair of mutants is demonstrated *in vitro* and *in vivo*, notably in mammalian cells. We hope that the availability of these mutants enables the specific and reliable targeting of Cre to asymmetric RT sites.

## MATERIALS AND METHODS

### Computational modeling and design

**Figure 2.**
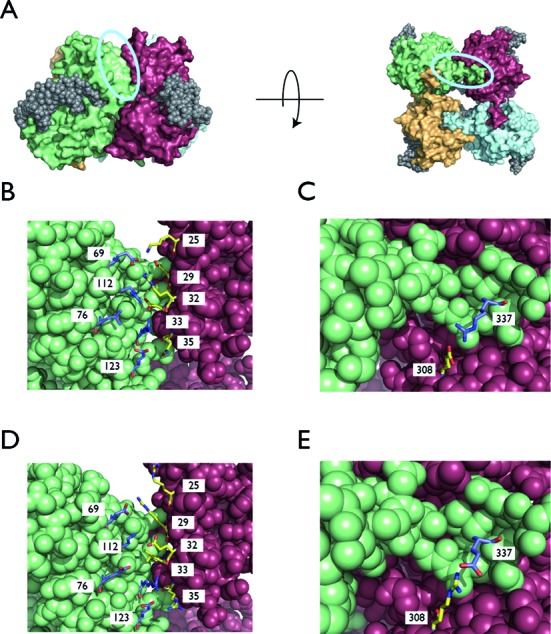
Mutated positions in the monomer–monomer interface. (**A**) The arrangement of Cre monomers on a loxP Holliday junction. The nucleic acid is shown as gray spheres and each Cre monomer is rendered in a separate color. The largest area of contact is indicated with a cyan oval on a side view of the complex (left side), and likewise the salt bridge that was inverted, shown in a bottom view (right side). (**B**) A set of interacting residues across the monomer–monomer interface was selected by eye for computational redesign (positions 25, 29, 32, 33, 35, 69, 72, 76, 119 and 123). The experimentally determined conformations of the side chains at these positions are shown (PDB code: 1KBU) (20)). In a third round of rational design, positions 35 and 123 were mutated to hydrophobic residues. (**C**) A putative salt bridge between a glutamate at position 308 and an arginine at position 337 is observed in the wild-type crystal structure. (**D**) The predicted model of the monomer–monomer interface after computational redesign is shown. The amino acids at positions 29 and 32 switch their electrostatic charge relative to wild-type, position 33 switches from charged to hydrophobic and positions 76 and 35 switch from uncharged to charged amino acids. (**E**) A putative model for the charge swap at positions 308 and 337 preserves a salt bridge, but with a change in polarity.

We selected the 2.2 Å crystal structure of a Cre-loxP Holliday junction as a template for computational design (PDB code: 1KBU (20)). The protein design capabilities of Rosetta3 (21) were used to select amino acids to form an alternative interface between Cre monomers. Amino acid positions 25, 29, 32, 33, 35 from chain A and 69, 72, 76, 119, 123 from chain B were chosen by eye for redesign because they form multiple interactions across the largest region of contact between monomers, but do not participate in the protein–DNA interface (Figure 2). At each of these positions, the calculation permitted mutation to a subset of amino acids including positive, negative or non-polar amino acids (AVMLDERK). The redesign calculation used the standard RosettaDesign fixed backbone algorithm. Sidechain rotamers were built using a backbone-dependent rotamer library (22). Extra rotamers sampling additional values for the χ_1_ and χ_2_ side chain torsion angles were included in the design calculation (command line options –ex1, –ex2 in Rosetta). A scoring function using a softened form of the Lennard-Jones potential (soft_rep_design (23)) was used to evaluate the interactions between the rotamers and the fixed backbone, and between rotamers at different positions. The combinatorial search through conformational space was accomplished using a Monte Carlo method with Metropolis acceptance criteria.

### Gene construction and protein expression

A gene encoding wild-type Cre recombinase with an N-terminal Met-His_7_ tag was constructed from 100 bp overlapping oligonucleotides ordered from Integrated DNA technologies (IDT) and cloned into the pET42a vector. Cre mutants were generated by site-directed mutagenesis. Proteins were expressed in BL21(DE3) star cells at 30°C using the autoinduction protocol of Studier (24). Details of the purification strategy are given in the Supplemental Materials.

### *In vitro* recombinase activity assay

Two direct loxP repeats or other variants of loxP/M7 sites separated by a ∼0.5 kb spacer were cloned between the XbaI and SphI sites of the pBAD33 plasmid. The 0.7 kb DNA substrate for *in vitro* recombination assays was generated by polymerase chain reaction (PCR) amplification with pBAD-forward and pBAD-reverse primers. One microgram of the DNA substrate was incubated with 1 μM Cre in 10 μl of 50 mM Tris–HCl, pH 7.8, 50 mM NaCl and 10 mM MgCl_2_ for 12 h at 37°C. The total amount of protein used is the same across all *in vitro* assays. Reactions were stopped by incubation at 98°C for 20 min. Reactions were analyzed on 2% agarose gels and visualized by staining with GelGreen nucleic acid stain (Biotium).

### Cell culture and transfection

The plasmid pGL4.23 (GenBank accession number: DQ904455) containing a multiple cloning site (MCS) for insertion of a response element of interest upstream of a minimal promoter and a gene encoding luc2 was purchased from Promega. The original minimal promoter in pGL4.23 was replaced with the haemoglobin beta (HBB) gene minimal promoter 144 bp upstream of the HBB transcription start site. The HBB minimal promoter has only the basic components for transcription (i.e. TATA box and GC box) and was amplified by PCR from mouse genomic DNA. The coding sequence of luc2 in PGL4.23 gene was replaced with different mutants of Cre recombinase using Gibson assembly. The enhancer candidates (CMV and SP1 enhancers) were then cloned into the MCS upstream of the minimal promoter. The engineered plasmids were isolated using standard molecular biology techniques and were confirmed by Sanger sequencing.

Ai14 mouse embryonic stem (ES) cells were engineered by targeted insertion of a construct containing the CAG promoter, followed by a floxed stop cassette-controlled red fluorescent marker gene (tdTomato) (Figure 4A) (25). The Ai14 mouse ES cells were cultured in complete media consisting of Dulbecco's modified eagle media (DMEM; Gibco) supplemented with 10% new born calf serum, 10% fetal bovine serum (FBS; Gibco) and 0.3 mM of each of the following nucleosides: adenosine, guanosine, cytosine, thymidine and uridine (Sigma-Aldrich). To maintain their undifferentiated state, the cells were also cultured in flasks coated with a 0.1% gelatin solution (Sigma-Aldrich) in the presence of 1000 U/ml leukemia inhibitory factor (LIF; Chemicon) and 20 mM β-mercaptoethanol (BME; Invitrogen).

Plasmids used for transfection of cells were prepared using EndoFree Plasmid Maxi Kits (Qiagen). About 2 × 10^5^ Ai14 ESCs were plated in one well of a 6-well plate 1 day prior to transfection with complete medium plus LIF in feeder free conditions. The cells were then transfected at 70% confluence by Lipofectamine 2000 (Invitrogen). For each transfection experiment, a total of 1 μg of plasmid DNA and 8 μl of Lipofectamine 2000 reagent were mixed following the manufacturer's protocol, and incubated at room temperature for 5 min before adding to the culture medium. The medium was replaced with fresh ESC medium plus LIF the following day and cells were cultured for another day before harvested for fluorescence activated cell sorting (FACS).

### Flow cytometry

Upon reaching ∼100% confluence, the cells were trypsinized from the plate and were suspended in Hank's Balanced Salt Solution (HBSS) supplemented with 2 mM EDTA, washed once with phosphate buffered saline (PBS) and resuspended in 500 μl PBS. Cellular fluorescence was analyzed on an iCyt Reflection HAPS2 cell sorter at the Washington University Siteman Flow Cytometry Core. Cells were treated with propidium iodide (2 μg/ml) prior to sorting to counter-select dead cells. The gate was set relative to cells transfected with plasmids lacking red fluorescent protein genes (negative controls) to eliminate non-specific background reporting. A minimum of 7000 total cells was analyzed from each FACS and post-sort analysis was performed with FlowJo software to obtain the percentage of tdTomato positive cells.

### Recombinase assay in mouse retinal explants

Electroporations and explant cultures were performed as previously described (26). Retinal explants were electroporated in a chamber containing 0.5 μg/ml each of supercoiled DNA encoding a gene for *Nrl*-eGFP as a control for electroporation efficiency, a reporter construct for Cre activity comprised of DsRed preceded by a floxed stop codon and a gene encoding either wild-type or engineered Cre under control of the *Nrl* promoter (27). Three replicates were performed for each electroporation. Quantification of fluorescence in retinal explants was accomplished using the ImageJ program (http://rsbweb.nih.gov/ij/) using a previously described protocol (28).

## RESULTS

### Computational redesign of a non-native but functional protein–protein interface between Cre recombinase monomers

We desired an engineered protein interface between Cre recombinase monomers that could form a functional complex, yet be incompatible with the wild-type interface. The two sides of such an interface could then be mixed with the other sides of the wild-type interface to yield two distinct Cre mutants. These mutants, by virtue of possessing incompatible interfaces, could not form functional homotetrameric complexes, but could be combined to form a functional heterotetramer (Figure 1D). We selected the 2.2 Å crystal structure of a Cre-loxP Holliday junction (PDB code: 1KBU) (20) as our template for computational design. We then selected the largest monomer–monomer interface patch for redesign, focusing on residues that did not participate in any contacts with DNA (cyan oval on left side of Figure 2A). We used the Rosetta molecular modeling program to redesign five residues on each side of the interface (see ‘Materials and Methods’ section), although in some cases (2 of 10) the wild-type amino acid was retained by the design calculation. The set of mutations that constitute the redesigned interface are the combined A1 and B1 mutations given in Table 1. When evaluated with the Rosetta full atom scoring function, the redesigned interface is 2.8 Rosetta units (RU) worse than the wild-type interface. Although the redesigned sequence was selected without regard for destabilizing mixed engineered/wild-type interfaces, models for the Cre–A1 and Cre–B1 homotetramer interfaces were predicted to be 9.8 and 20.49 RU worse than wild-type, respectively. Inspection of individual terms shows that the Cre–A1 and Cre–B1 models possess interface residues with side chains in strained conformations, presumably due to lack of favorable interactions.

**Table 1.** Mutations for each round of protein engineering

Cre mutant	Mutations
Cre–A1	K25R, D29R, R32E, D33L, Q35R
Cre–B1	E69D, R72K, L76E
Cre–A2	Cre–A1 + R337E
Cre–B2	Cre–B1 + E308R
Cre–A3	Cre–A2 + E123L

**Figure 3.**
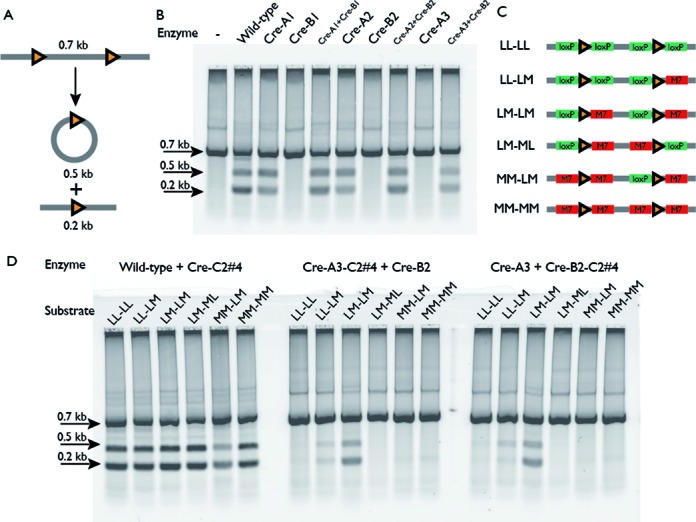
*In vitro* recombination assay of Cre mutants. (**A**) *In vitro* recombinase assay. A 0.7 kb linear DNA substrate with direct repeats of the loxP site (orange triangles) is incubated with wild-type or mutant Cre recombinase. The activity of functional Cre complexes results in production of a 0.5 kb circular product and a 0.2 kb linear product through intra-molecular excision. (**B**) *In vitro* assay results. Lane 1: DNA substrate alone; lane 2: wild-type Cre; lanes 3–5: first round redesigned Cre mutants Cre–A1, Cre–B1 and a mixture of the two (CreA1 + CreB1); lanes 6–8: second round redesigned Cre mutants Cre–A2, Cre–B2 and a mixture of the two (Cre–A2 + Cre–B2); lanes 9–10: third round mutant Cre–A3 and a mixture of Cre–A3 + Cre–B2. All Cre–B mutants are inactive in isolation. Cre–A mutants progressively lose homotetramer activity through the three rounds of design. (**C**) *In vitro* substrates for asymmetric recombination target site experiments. RT half-sites in the linear DNA substrate described in panel (A) were systematically varied to incorporate the M7 sequence (13). LoxP and M7 half-sites are rendered as green and red boxes, and abbreviated by the letters L and M, respectively. Combinations ranged from entirely loxP (LL–LL, the same as in panel (A)) to entirely M7 (MM–MM), including hybrid RT sites situated as both direct (LM–LM) and inverted (LM–ML) repeats. (**D**) The effect of controlled assembly of heterotetrameric Cre complexes. Each of the mixed loxP/M7 substrates was incubated with a pair of recombinases, one with mutations that recognize the M7 RT halfsite (Cre–C2#4) and the other with preference for the loxP half-site. In the left panel, the two proteins have no additional mutations to control complex formation. In the middle and right panels, recombinases with different RT specificities are combined with the Cre–A3 and Cre–B2 mutations, with both possible combinations tested. The restriction of permissible substrates by the Cre–A3 and Cre–B2 mutations are consistent with a requirement for an (ABAB) heterotetramer to achieve recombinase activity.

We tested the redesigned interface by generating pairs of Cre mutants such that each mutant possesses one side of the interface, with the other side fixed as wild-type. We assayed members of each pair for recombinase activity *in vitro* both individually and in combination (Figure 3). While the combined pair of redesigned mutants was active (Cre–A1 + Cre–B1 in Figure 3B; see Table 1 for mutations), one of the mutants (Cre–A1) was active individually, indicating that this hybrid redesign/wild-type interface was functionally compatible, in violation of our negative engineering goal (Figure 1D).

### Iterative rounds of rational design enhance the formation of (ABAB) complexes

We attempted to find another region of contact between monomers in the Cre complex that we could mutate in an attempt to further destabilize homotetrameric Cre–A1 complexes. Visual inspection of the Cre crystal structure revealed a salt bridge between Glu308 and Arg337 (Figure 2C) that we hypothesized could be inverted to obtain additional specificity for the heterotetramic complex (Figure 2E). We therefore further mutated Cre–A1 (adding R337E) to yield Cre–A2 and mutated Cre–B1 (adding E308R) to yield Cre–B2. Thus, homotetrameric complexes of Cre–A2 would place two glutamate residues at 308 and 337 in close proximity, and Cre–B2 would likewise pair two arginine residues, yielding unfavorable electrostatic repulsion in either case. Our *in vitro* recombinase assay showed that the Cre–A2 + Cre–B2 combination exhibited robust recombinase activity. However, while its activity is reduced relative to Cre–A1, the Cre–A2 monomer was still capable of forming a functional homotetrameric complex (Figure 3B).

We selected a polar interaction between monomers as the final site for mutagenesis. We hypothesized that a replacement interaction consisting of hydrophobic residues would be incompatible with the pre-existing polar interaction. Structural modeling suggested that the mutation E123L and Q35L could create a tight packing interaction between leucine residues across the monomer-monomer interface, but that interfaces combining a polar residue from the wild-type interface with either leucine from the engineered interface would be energetically unfavorable.

*In vitro* assays indicated that the E123L mutation did indeed penalize formation of functional homotetrameric complexes, but that the Q35L mutation unexpectedly facilitated homotetramer formation in the previously inactive B2 mutant (data not shown). Consequently, we applied the E123L mutation to Cre–A2 to create Cre–A3. This mutation successfully disrupted formation of Cre–A2 homotetramers while preserving activity in the Cre–A3 + Cre–B2 heterotetramer (Figure 3B). The improvement in specificity appears to come from selective destabilization of the Cre–A3 homotetramer with limited destabilization of the heterotetramer. The reactivation of Cre–B1 by the Q35L mutation is especially puzzling, as the distance between the α carbon of position 35 and that of the nearest mutated position from Cre–B1 (position 76) is roughly 13 Å, suggesting that this mutation cannot directly alleviate the interface deficiencies introduced by the Cre–B1 mutations. This mutation may allow for a subtle rearrangement of the charged side chains at the interface that yields a functional complex through a mechanism that requires introduction of modes of relaxation that are not captured by our model.

To test whether our round 1 mutations are essential to enforce heterotetramer formation, we generated Cre mutants with only round 2 and round 3 mutations. The salt-bridge swap from round 2 alone yields two Cre mutants with reduced but clear activity (data not shown). We combined round 2 and round 3 mutations to create Cre–E123L–E308R and Cre–E123L–R337E. *In vitro* assays indicated that these mutants do not form an obligate heterotetrameric pair (Supplemental Figure S1A). We conclude that the combined effects of mutations from all three rounds are necessary to achieve our design goal.

### Heterotetrameric mutations can be combined with DNA specificity altering mutations to enhance target site specificity

We hypothesized that the ability to control the assembly of functional Cre complexes would lead to higher fidelity recognition of asymmetric RT sites if used in combination with recombinases with different DNA specificities. Directed evolution has already been exploited to generate mutants of Cre recombinase that can utilize altered RT sites. A mutant (termed Cre–C2#4) with five amino acid mutations relative to wild-type has been shown to recombine an alternate RT site termed loxM7 (13). The monomer–monomer interface mutations from Cre–A3 and Cre–B2 were applied separately to the Cre–C2#4 mutant. If the proteins with different DNA specificities exhibit the expected ABAB heterotetrameric pattern assembly, they should only recombine DNA half-sites with a specific spatial arrangement, yielding enhanced target specificity.

To this end, we designed DNA substrates harboring direct repeats of six different loxP/M7 hybrid RT sites as a rigorous test of specificity (Figure 3C). We expect that a mixture of wild-type Cre and Cre–C2#4 (both of which lack our obligate heterotetrameric mutations) could recombine all of the six RT sites, as the individual monomers can combine in any manner dictated by the sequences of the RT half-sites. In contrast, a combination of the designed Cre–A3–C2#4 and Cre–B2 recombinases, or similarly the Cre–A3 and Cre–B2–C2#4 recombinases, would specifically recombine the LM–LM site, but not the other five RT sites (Figure 3C). This would imply that heterotetrameric Cre mutants will have less off-target activity when used for genome editing.

*In vitro* assays confirmed that the heterotetrameric Cre is more specific in recombining different arrangement of loxP/M7 sites (Figure 3D). Cre–C2#4 is slightly promiscuous, and can recombine loxP sites when incubated with DNA substrate for a long period of time ((13), Supplemental Figure S1B). The observed partial activity of the two designed pairs on LL-ML site (lane 2 in the middle and right gels of Figure 3D) is most likely the result of the promiscuity of Cre–C2#4's DNA specificity. It is also interesting to note that, because the four Cre monomers work cooperatively to recombine the DNA target, wild-type Cre and Cre–C2#4 homotetramers recombined most of the loxP/M7 hybrid sites on their own ((29), Supplemental Figure S1B). The specificity shown here by the two designed pairs provides strong evidence that our mutant recombinases indeed form an ABAB heterotetrameric complex.

### Obligate heterotetramer formation is preserved in mammalian cells

**Figure 4.**
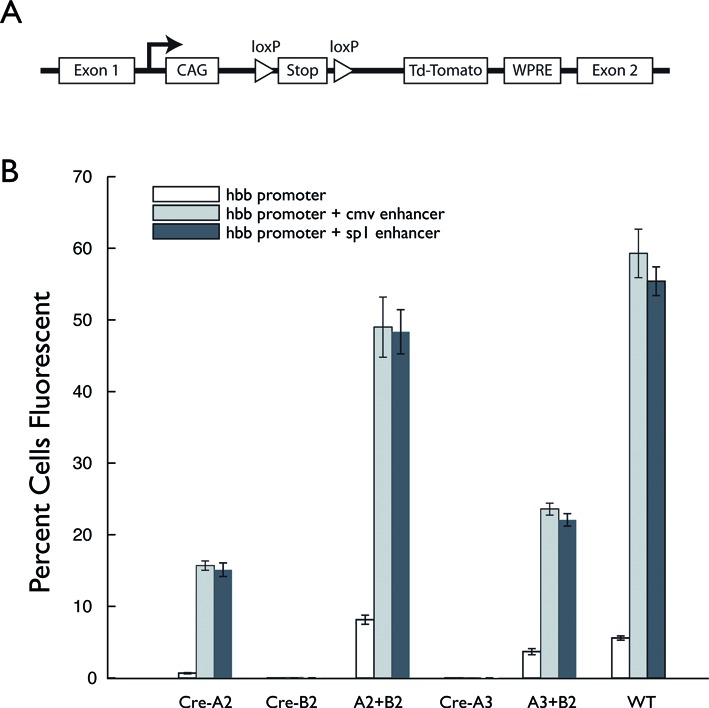
Cre mutant pair recapitulates requirement for heterotetramer formation in mouse ES cell cultures. (**A**) Diagram of the Cre-reporter cell line. The Cre-reporter cassette was inserted into the Rosa26 locus, in the intron between endogenous exons 1 and 2. In the cassette, a gene encoding tdTomato is preceded by a floxed stop codon and followed by the woodchuck post-transcriptional regulatory element (WPRE). (**B**) Plasmids with the hbb minimal promoter driving expression of different Cre variants either alone or augmented with the CMV or SP1 enhancers were co-transfected into Ai14 mouse embryonic stem (ES) cells containing a fluorescent reporter cassette. The same total amount of DNA was used for all transfections and three independent transfections were performed for each Cre variant. The percent of tdTomato positive cells was measured by flow cytometry. A total of 7000 cells were sorted after each transfection. The average number of tdTomato positive cells for each Cre variant or combination of variants is shown. For Cre–B2 and Cre–A3, cell counts were less than five (out of 7000) for all promoter constructs (Supplemental Table S1).

We envision RMCE in mammalian cells as the target application for our heterotetramer-forming Cre mutants. We employed two reporter systems to determine whether the engineered proteins satisfy our design goals in mammalian cells. First, we assayed the recombinase activity of the Cre mutants in a mouse ES cell reporter line by flow cytometry. We inserted a gene for the tandem dimer tomato (tdTomato) fluorescent protein downstream of a floxed stop codon at the *rosa26* locus (Figure 4A). Constructs encoding genes for the Cre mutants driven by the HBB minimal gene promoter, either alone or in combination with one of two enhancers (see ‘Materials and Methods’ section), were transfected into the reporter line and the cells expressing tdTomato were quantified by flow cytometry (Figure 4B, Supplemental Table S1).

Similar to the *in vitro* results, we observed the Cre–A 2 + Cre–B2 combination to be functional, while the Cre–A2 mutant retains significant activity as a homotetramer. Combining Cre–A3 with Cre–B2 yielded a suitable obligate heterotetrameric pair, retaining roughly 40% of wild-type Cre activity. Neither the Cre–A3 nor the Cre–B2 mutants exhibited appreciable activity alone.

**Figure 5.**
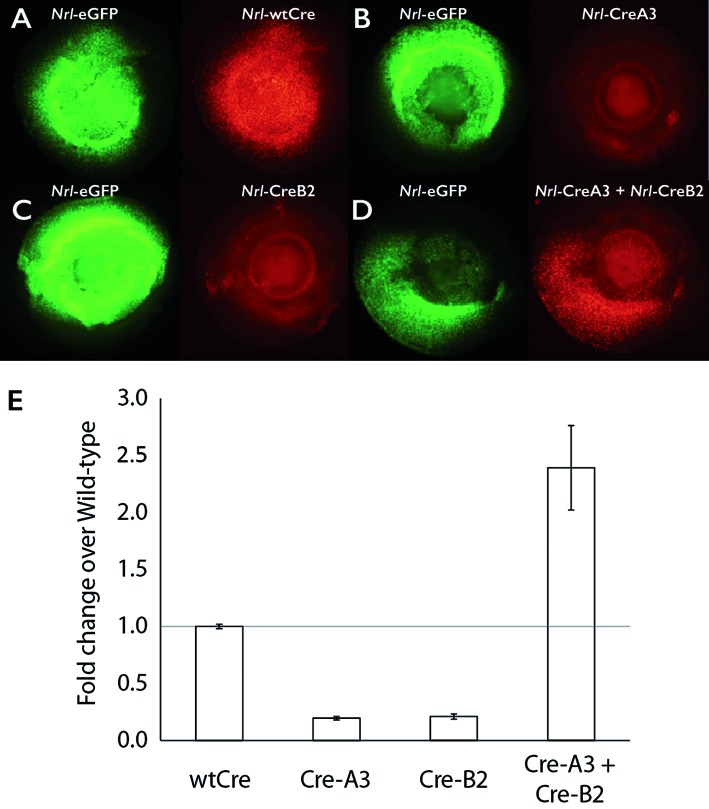
Engineered Cre mutants retain preference for heterotetrameric complex in mouse retinal cells. Dissected newborn mouse retinas (with lens in place) were electroporated with constructs encoding: (i) *Nrl*-eGFP as a control for electroporation efficiency, (ii) a reporter construct for Cre activity comprised of DsRed preceded by a floxed stop codon and (iii) a gene encoding either wild-type (**A**) or engineered Cre (**B**–**D**) under control of the *Nrl* promoter. The left side of each panel shows the fluorescence from the green channel, which indicates cells that were successfully electroporated. Fluorescence from the red channel results from removal of the floxed stop codon, indicating Cre activity. The lens shows some autofluorescence which is apparent as a central circular region of red fluorescence in B, C and D. (**E**) Quantification of activity of electroporated constructs relative to wild-type Cre.

We also evaluated the activity of the Cre mutants in mouse retinal explants. Dissected newborn mouse retinas were electroporated with a construct expressing green fluorescent protein (GFP) under the control of the rod photoreceptor-specific *Nrl* promoter (27) (as a loading control), Cre mutants under the control of the same *Nrl* promoter and a floxed tdTomato reporter construct. After eight days in explant culture, the retinas were harvested and imaged. The appearance of the flat-mounted retinas under epifluorescent illumination is shown in Figure 5. GFP fluorescence indicates areas of successful electroporation, and red fluorescence reports recombinase activity. Wild-type Cre shows robust activity, with all green cells also exhibiting red fluorescence (Figure 5A). The Cre–A3 and Cre–B2 mutants alone show very little activity (Figure 5B and C), while combining the two restores robust activity (Figure 5D). Quantification confirms that Cre–A3 and Cre–B2 form an obligate heterotetrameric pair in photoreceptor cells (Figure 5E).

## DISCUSSION

We sought to engineer a pair of mutants of Cre recombinase that form an obligate ABAB heterotetrameric complex. The Cre–A3 and Cre–B2 mutants are the result of an iterative process of computational and rational protein engineering. We have shown that the two mutants are inactive in isolation, but are functional when combined. Furthermore, we have shown that when additional mutations are used to confer an altered DNA specificity upon either one of the mutants, the arrangements of half-sites that are recombined are consistent with the formation of an ABAB complex. Although our attempts to confirm the composition of the functional complex directly via crystallography were unsuccessful, our data are strongly suggestive that we have succeeded in our goal.

Engineering a novel interface for Cre recombinase monomers that is incompatible with the wild-type interface involves two distinct requirements, one positive and one negative. The positive requirement is that the novel interface must give rise to a functional tetrameric complex. The negative requirement is that any combination of wild-type and engineered monomer surfaces must be functionally incompatible. We found that the negative engineering goal was more difficult to achieve. We were able to generate a novel functional interface using straightforward computational protein design. However, the mutations on one side of the interface (the Cre–A1 mutations) were still compatible with the wild-type residues on the other side. We found that additional rounds of rational design were required to reduce the residual activity of homotetrameric complexes.

A previous effort to create a heterotetrameric Cre complex identified concerted small-to-large and large-to-small hydrophobic mutations in an expression library that combinatorially mutagenized three tightly coupled residues (19). The engineered interface was functional, but one of the mutant surfaces retained significant activity in complex with the complementary wild-type surface. Perhaps unsurprisingly, a small-to-large mutation was incompatible with the wild-type surface, presumably due to steric clash. However, large-to-small mutations exhibited reduced activity relative to wild-type, likely a consequence of creating a cavity that destabilized, but did not destroy, the integrity of the interface. Although our mutations were selected to drive heterotetramer formation primarily based upon electrostatics rather that sterics, we ascribe our elimination of homotetramer activity to the increased number of residues and contact regions that we altered rather than the nature of the interactions that were altered or introduced.

Researchers have successfully demonstrated a different strategy for partitioning Cre recombinase into two variants that are only active when combined. It has been shown that Cre recombinase can be split into N- and C-terminal fragments (split-Cre) that can reconstitute a functional complex when co-expressed *in vivo* by virtue of coiled coil dimerization tags appended to each fragment (30). The motivation for this approach was to place the split-Cre fragments under different promoters, yielding enhanced control over the cell types in which functional Cre complexes are present and resulting in highly specific conditional gene regulation. However, this approach to splitting Cre is not suitable for our purpose of combining monomers with different DNA specificities. Each split–Cre complex retains specificity for the loxP RT site. Even if specificities of the DNA-contacting regions are altered, the assembly of N and C-terminal fragments is uncontrolled, allowing for multiple combinations of half-site RT site specificities (16) and making this decomposition unsuitable for targeting asymmetric sites with high specificity.

CRISPR-based systems have emerged as an attractive tool for genome engineering due to the ease with which the Cas9 nuclease can be redirected to arbitrary targets (31–33). CRISPR/Cas technology represents the logical conclusion of modular DSB inducing agents, as the Cas9 nuclease can be targeted to any site that contains a protospacer adjacent motif sequence (typically 3–5 bases in length) without mutating the protein itself. In cell culture, this activity can drive the efficient generation of loss-of-function mutants when the DSB is repaired by non-homologous end-joining (NHEJ), or gene conversion when homology-directed repair occurs in the presence of an exogenously provided repair template (34). Given these features of CRISPR/Cas systems, what role can mutants of Cre recombinase play in genome engineering applications?

Gene conversion by RMCE possesses advantages over DSB-induced gene conversion that are unique to enzymatically autonomous recombinases. A crucial advantage is that no other cofactors or endogenous cellular machinery are necessary. In particular, this avenue for genome editing does not rely upon the homology-directed DNA repair (HDR) system. The balance between DNA repair via HDR and via NHEJ is highly dependent on cell type and HDR itself is not a significant route for DNA repair in cells that are not replicating (3,4). Thus, RMCE approaches may prove to be the only effective route to gene conversion for postmitotic cells, where DSB-induced HDR performs poorly. Furthermore, DSB-stimulated gene conversion is efficient over a relatively short range (∼100 bp) (35). In contrast, cassette-mediated exchange is capable of correcting any mutation that falls within the RT site boundaries. Using RMCE, genetic intervals of >100 kb of DNA have been exchanged, with the size of the interval limited by the size of the donor construct, and not by the method itself (36).

The disadvantage of targeting mutant recombinases to endogenous sites in a genome is the difficulty with which recombinase DNA specificity is altered. While directed evolution has proven to be successful in generating novel RT specificities, the compatibility of DNA specificity altering mutations with our interface mutations is a concern. Our results show that in at least one case (loxM7) the mutations that alter DNA specificity are compatible with our mutations for controlling tetramer assembly. With respect to obtaining novel specificity Cre mutants, there is no realistic hope for any retargeting strategy that can rival the speed and ease of retargeting in CRISPR/Cas systems. We anticipate that endogenous site RMCE will be useful when a particular genomic locus is of sufficient interest to merit the effort required to obtain mutant recombinases whose RT specificities bracket the locus, or when there is a need to repeatedly exchange the DNA within the genetic interval. This may be the case when a locus harbors a large number of disease­-associated polymorphisms that span several kb, or when a ‘promoter bashing’ experimental approach is desired in an endogenous context.

We have presented an obligate heterotetrameric pair of Cre recombinase mutants. We have demonstrated that this pair can be used to form functional complexes that can recognize asymmetric RT sites. However, to realize the RMCE approach with maximal control over Cre complex formation, we will require a second pair of recombinase monomers to target the second asymmetric RT site that brackets the genetic cassette. This may be accomplished by engineering two additional Cre monomers that form a second obligate heterotetramer that is incompatible with the mutants we have described here. As this involves a large number of positive and negative constraints on monomer association, we suggest that an easier approach will be to use the knowledge of interacting residues we have identified in this study to direct rational redesign of the interface of a Cre homolog (37–39). Although no crystal structures are available for close homologs of Cre, sequence homology between recombinases has been recognized that could assist in generating obligate heterotetrameric mutants (37,40). We are currently investigating the feasibility of this approach.

## Supplementary Material

SUPPLEMENTARY DATA
