# Supplementary material for: Redesign of the monomer–monomer interface of Cre recombinase yields an obligate heterotetrameric complex
Source: Nucleic Acids Res. 2015 Oct 10;43(18):9076–85. doi: 10.1093/nar/gkv901 (PMC4605323; doi:10.1093/nar/gkv901)
Supplement: SUPPLEMENTARY DATA [file supp_43_18_9076__index.html]

Redesign of the monomer–monomer interface of Cre recombinase yields an obligate heterotetrameric complex — Redesign of the monomer–monomer interface of Cre recombinase yields an obligate heterotetrameric complex — SUPPLEMENTARY DATA 

# Redesign of the monomer–monomer interface of Cre recombinase yields an obligate heterotetrameric complex

## SUPPLEMENTARY DATA

- SUPPLEMENTARY DATA
