# Supplementary material for: Redesign of the monomer–monomer interface of Cre recombinase yields an obligate heterotetrameric complex
Source: Nucleic Acids Res. 2015 Oct 10;43(18):9076–85. doi: 10.1093/nar/gkv901 (PMC4605323; doi:10.1093/nar/gkv901)
Supplement: SUPPLEMENTARY DATA [file supp_gkv901_nar-01405-h-2015-File008.pdf]

Supplementary Materials for

Redesign of the monomer-monomer interface of Cre recombinase yields an obligate heterotetrameric complex

Chi Zhang[1], Connie A. Myers[2], Zongtai Qi[3], Robi D. Mitra[4],

Joseph C. Corbo[2], and James J. Havranek[5]

[1]Program in Computational and Systems Biology, [2]Department of Pathology and Immunology, [3]Program in Molecular Genetics and Genomics, [4]Department of Genetics, and [5]Department of Biochemistry and Molecular Biophysics

Washington University in St. Louis, St. Louis, MO 63110

Supplemental Methods

Protein purification of Cre recombinase variants. Proteins were expressed in BL21(DE3) star cells at 25°C using the autoinduction protocol of Studier {Studier, 2005, Protein Expr Purif, 41, 207-234}. The cells were harvested by centrifugation after 48 hours. The cell paste was resuspended in 25mL buffer A (0.7M NaCl, 50mM Tris-HCl pH7.8, 5mM Imidazole), lysed by sonication on ice, and separated from cellular debris by centrifugation. The filtered supernatant was applied to a HisTrap™ HP column (Amersham) and washed with 30mL Buffer A. The column was then washed with 20mL 15% buffer B (0.7M NaCl, 50mM Tris-HCl pH7.8, 500mM Imidazole). Cre was eluted with a linear gradient from 15% buffer B to 100% buffer B, with the elution peak starting at roughly 20% buffer B. Approximately 10mL of the eluted protein was collected and dialyzed overnight at 4°C against 5L dialysis buffer (0.7 M NaCl, 50mM Tris-HCl pH7.8). The protein concentration was then determined by UV absorbance using an extinction coefficient at 280nm of 49 $mM^{-1}cm^{-1}$. The protein retained activity for months when stored at 4° C.

Supplemental Table 1. Cell sorting data from mouse ES cells

		Replicate		
		1st	2nd	3rd
total # of cells sorted		7000	7000	7000
Cre-A1	hbb	414	378	391
	hbb+cmv	3852	3528	3687
	hbb+sp1	3750	3419	3501
Cre-B1	hbb	97	102	85
	hbb+cmv	1237	1258	1120
	hbb+sp1	1150	1080	1202
A1+B1	hbb	1117	1212	1324
	hbb+cmv	5866	6029	6358
	hbb+sp1	5702	6121	5987
Cre-A2	hbb	47	52	41
	hbb+cmv	1127	1116	1052
	hbb+sp1	1053	1002	1119
Cre-B2	hbb	0	0	1
	hbb+cmv	2	2	4
	hbb+sp1	2	1	3
A2+B2	hbb	573	528	607
	hbb+cmv	3180	3409	3698
	hbb+sp1	3221	3336	3593
Cre-A3	hbb	0	0	1
	hbb+cmv	0	1	1
	hbb+sp1	1	0	0
A3+B2	hbb	256	233	284
	hbb+cmv	1598	1652	1701
	hbb+sp1	1503	1527	1606
WT	hbb	372	391	408
	hbb+cmv	3914	4223	4312
	hbb+sp1	3815	3799	4021

Plasmids with the hbb minimal promoter alone or with either the cmv and sp1 enhancers driving different cre variants were co-transfected into Ai14 mouse embryonic stem (ES) cells containing a reporter cassette with tdTomato preceded by a floxed stop codon. The same total amount of DNA was used for all transfections, and 3 independent transfections were performed for each Cre variant. The number of tdTomato positive cells was measured by flow cytometry.

Supplemental Figure 1.

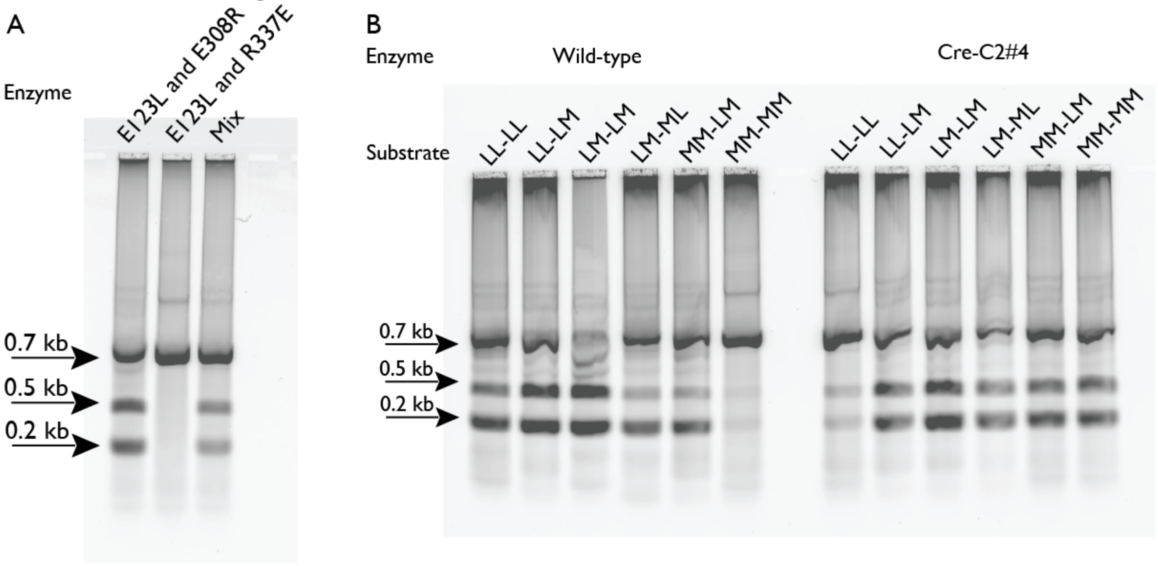

Supplemental Figure 1 Legend. (A) *In vitro* assay results for Cre mutant pairs lacking computationally designed mutations. Linear DNA substrate (0.7 kb) with direct loxP repeats was incubated with Cre mutants. Lane 1: Cre-E123L/E308R; lane 2: Cre-E123L/R337E; lane 3: A 1:1 mixture of above two Cre mutants. The E123L/E308R mutations are insufficient to eliminate activity in this monomer, indicating that additional mutations are necessary to achieve the goal of obligate heterotetramers. (B) *In vitro* assay results for Cre proteins with wild-type monomer-monomer interfaces. Wild-type Cre and Cre-C2#4 were assayed for recombination activity against six loxP/M7 hybrid RT sites. The left panel: wild-type Cre recombined robustly on all six RT sites except for all M7 site. The right panel: Cre-C2#4 recombined all six RT sites, although with diminished activity with increased number of loxP half-sites.

Supplemental Figure 2.

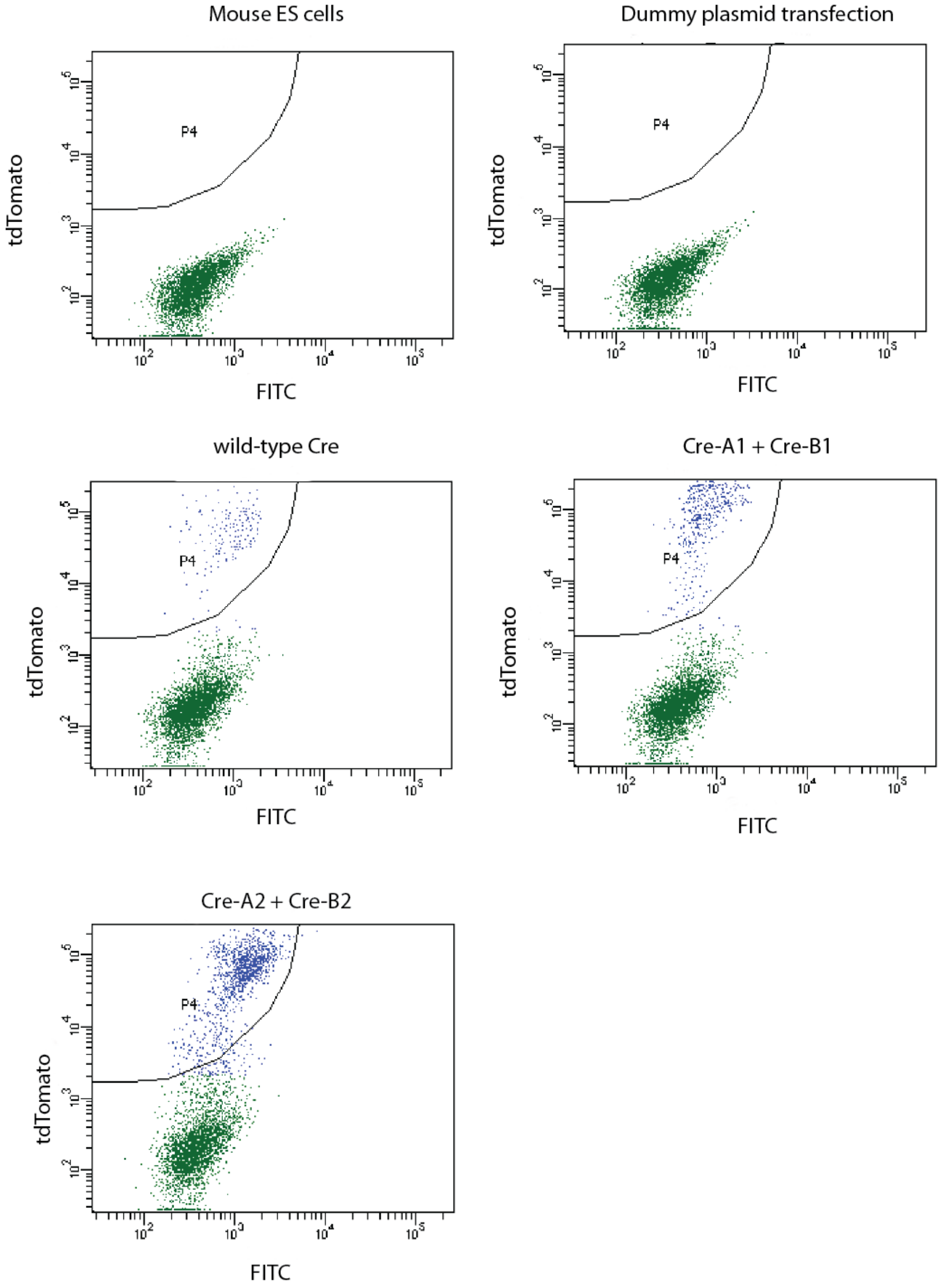

Supplemental Figure 2 Legend. Representative raw data from flow sorting experiments. Each point shows the fluorescence in the red channel (tdTomato)

versus green channel (FITC). The cell-only and dummy plasmid experiments exhibit roughly identical autofluorescence. The gating for identifying RFP-positive is the region of each plot labeled ‘P4’.
